# Physical multimorbidity and loneliness: A population-based study

**DOI:** 10.1371/journal.pone.0191651

**Published:** 2018-01-24

**Authors:** Andrew Stickley, Ai Koyanagi

**Affiliations:** 1 The Stockholm Center for Health and Social Change (SCOHOST), Södertörn University, Huddinge, Sweden; 2 Parc Sanitari Sant Joan de Déu, Universitat de Barcelona, Fundació Sant Joan de Déu, Sant Boi de Llobregat, Barcelona, Spain; 3 Instituto de Salud Carlos III, Centro de Investigación Biomédica en Red de Salud Mental, CIBERSAM, Madrid, Spain; Cardiff University, UNITED KINGDOM

## Abstract

Multimorbidity has been linked to a variety of negative outcomes although as yet, there has been little research on its association with loneliness. This study examined the association between physical multimorbidity (≥ 2 physical diseases) and loneliness in the general population and its potential mediators. Data came from the Adult Psychiatric Morbidity Survey 2007 (N = 7403, aged ≥16 years). Information was obtained on 20 doctor diagnosed physical conditions that were present in the previous year. An item from the Social Functioning Questionnaire (SFQ) was used to obtain information on loneliness. Multivariable logistic regression analysis was used to examine associations. An increasing number of physical diseases was associated with higher odds for loneliness. Compared to no physical diseases, the odds ratio (OR) (95% confidence interval: CI) for loneliness increased from 1.34 (1.13–1.59) to 2.82 (2.11–3.78) between one and ≥5 physical diseases. This association was particularly strong in the youngest age group (i.e. 16–44 years). The loneliness-physical multimorbidity association was significantly mediated by stressful life events (% mediated 11.1%-30.5%), anxiety (30.2%), and depression (15.4%). Physical multimorbidity is associated with increased odds for loneliness. Prospective research is now needed to further elucidate this association and the factors that underlie it.

## Introduction

Multimorbidity (the presence of 2 or more chronic/acute diseases) is common in the general population [1]. A recent study that used World Health Survey data from 27 low- and middle-income countries (LMICs) and 1 high-income country (HIC) showed that the prevalence of multimorbidity ranged from 1.7% (Myanmar) to 15.2% (Nepal) and averaged 7.8% across all LMICs countries [2]. Although research has indicated that the prevalence of multimorbidity increases with age, it is observed in all adult age groups [2,3]. This is alarming as the co-occurrence of chronic disease has been associated with a variety of negative outcomes including worse quality of life [4], poorer physical function [5], greater health care use [6], as well as an increased risk for premature mortality [7].

The current study will examine physical multimorbidity—the co-occurrence of 2 or more physical diseases. Although there has been increased attention on this phenomenon recently [8–11], there are still many gaps in our knowledge. For example, relatively little is known about the association between physical multimorbidity and loneliness, that is, “the unpleasant experience that occurs when a person’s network of social relations is deficient in some important way, either quantitatively or qualitatively” [12]. This may be an important omission. Loneliness is not only common in the general population with studies showing that on average, approximately 5–15% of people report often feeling lonely [13–16], but there is some evidence that loneliness might be a significant factor for health in its own right. Specifically, feeling lonely has been linked to an increased risk for a variety of adverse health outcomes including premature mortality [17–19].

Importantly, previous research has indicated that there might be an association between physical disease and loneliness. In particular, an earlier study found that there was an increased risk for loneliness in individuals with chronic diseases [20]. Other studies have also shown an association between feeling lonely and heart disease, hypertension, stroke [21–23] and Alzheimer disease [24]. However, this research has also indicated that not all physical diseases are linked to loneliness and that the association between loneliness and specific diseases may vary between different population subgroups [21].

Despite these studies linking physical disease and loneliness, as yet, there has been comparatively little research on the association between physical multimorbidity and loneliness. Moreover, to the best of our knowledge, the few studies undertaken to date on the association between the number of illnesses/multimorbidity and loneliness or vice versa have been confined to middle-aged and older adults (age ≥ 45) and produced mixed results [25–29]. Specifically, while studies among adults aged 52–92 in Denmark, 65 and above in the United States and 75 and above in Israel respectively linked the number of chronic illnesses and greater comorbidity to loneliness [25,27,28], a study among veterans aged 60 and above in the United States found that a bivariate association between the number of medical conditions and loneliness became non-significant in a stepwise linear regression analysis [29]. In addition, research undertaken among men and women aged 45 and above in Canada and Australia found that multimorbidity was significantly linked to loneliness in all age-sex groups except among Australian women aged 75 and above [26].

Given the absence of research on the association between (physical) multimorbidity and loneliness among adults of all ages and the mixed findings from research that has focused on the multimorbidity-loneliness association in middle-aged and older adults, the current study had two aims: (1) to examine the association between physical multimorbidity and loneliness in a general population sample; and (2) to determine if any factors might be important for this association.

## Materials and methods

### Study population

This study used data from the Adult Psychiatric Morbidity Survey (APMS) 2007 (N = 7403). Details of the survey have been published elsewhere previously [30]. In brief, the survey was conducted in England between October 2006 and December 2007 by the National Center for Social Research and Leicester University. To obtain a nationally representative sample of the adult population aged ≥16 years old residing in private households, multistage stratified probability sampling was used. The small user Postcode Address File (PAF) served as the sampling frame with postcode sectors serving as the primary sampling units (PSUs). Sectors were stratified by both region and socioeconomic status. One person was randomly selected from each randomly selected household. The survey response rate was 57% (respondents from 7461/13171 eligible households agreed to participate). To correct for survey non-response, sampling weights were generated to ensure that the sample was representative of its intended target population. Details of the weighting procedure are provided in the survey report [30]. The Royal Free Hospital and Medical School Research Ethics Committee provided ethical approval for the study. The survey methodology was carried out in accordance with the relevant guidelines and regulations with all participants providing written informed consent.

### Data availability

The data used in this study are third party data (i.e., they are not owned and were not collected by the authors) that have been made publicly available by the National Center for Social Research via the UK data archive. The authors did not have any special access privileges and other authors can access these data in the same manner as the authors in this study did i.e. registration is required and standard conditions of use apply. Details of how to access the Adult Psychiatric Morbidity Survey 2007 dataset are available at: https://discover.ukdataservice.ac.uk/catalogue/?sn=6379

### Main measures

#### Physical illnesses

A question which enquired about the presence of 20 physical health conditions was used to assess physical illnesses (cancer, diabetes, epilepsy, migraine, cataracts/eyesight problems, ear/hearing problems, stroke, heart attack/angina, high blood pressure, bronchitis/emphysema, asthma, allergies, stomach ulcer or other digestive problems, liver problems, bowel/colon problems, bladder problems/incontinence, arthritis, bone/back/joint/muscle problems, infectious disease, and skin problems). To be counted, conditions had to have been diagnosed by a doctor or other health professional and have been present in the previous 12 months. The number of physical diseases was summed and categorized as 0, 1, 2, 3, 4, and ≥5. Multimorbidity was defined as two or more physical diseases [31].

#### Loneliness

This was measured with one item from the Social Functioning Questionnaire (SFQ) [32]. Respondents were asked to assess to what extent they had felt ‘lonely and isolated from other people’ in the previous two weeks. The answer options were ‘very much’, ‘sometimes’, ‘not often’, and ‘not at all’. In the current study, response options were dichotomized with those who answered ‘sometimes’ and ‘very much’ being categorized as lonely [33].

### Covariates

#### Smoking

The question, ‘Have you ever smoked a cigarette?’ with ‘yes’ or ‘no’ answer options was used to assess smoking status.

#### Alcohol dependence

This was assessed with two instruments. The Alcohol Use Disorders Identification Test (AUDIT) was firstly used to assess alcohol consumption [34]. When a respondent’s AUDIT score was ≥ 10 they were also assessed for alcohol dependence using the Severity of Alcohol Dependence Questionnaire (SADQ-C) [35]. Those who scored ≥ 4/60 on this measure were categorized as having past 6-month alcohol dependence.

#### Drug use

Information was obtained on the past year use of the following drugs: cannabis, amphetamines, cocaine, crack, ecstasy, heroin, acid or LSD, magic mushrooms, methadone or physeptone, tranquilizers, amyl nitrate, anabolic steroids, and glues. Respondents taking any of these drugs were categorized as past 12-month drug users.

#### Disordered eating

Possible eating disorder was assessed with five items from the SCOFF eating disorder screening tool [36]. Respondents were asked whether, in the past year, they (1) had lost more than one stone (6.35kg) in 3 months; (2) had made him/herself be sick because he/she felt uncomfortably full; (3) had worried that he/she had lost control over how much he/she eats; (4) believed [himself/herself] to be fat when others said that he/she was too thin; and (5) thought that food dominated his/her life. Using yes/no response options, a positive screen was categorized as two or more affirmative answers [36].

#### Obesity

Self-reported weight and height data were used to determine body mass index (BMI), calculated as weight in kilograms divided by height in meters squared. As a preliminary examination of the data revealed that only extreme levels of obesity were associated with loneliness, we used ≥35 kg/m^2^ (obesity class I) as the cut-off.

#### Stressful life events

Seventeen questions on the lifetime occurrence of experiences such as the death of a family member, financial crises, sexual abuse etc., were used to assess stressful life events (S1 Table). Two different stressful life events measures were constructed depending on whether the event last occurred when the respondent was <16 or ≥16 years of age. For the latter measure, a summed total number of stressful life events was based on all 17 questions, while 8 age-appropriate potentially stressful life events were assessed before age 16 (all of which had a prevalence of at least 2%) (S1 Table).

#### Depressive episode and anxiety disorders

The Clinical Interview Schedule Revised (CIS-R), was used to identify non-psychotic symptoms in the prior week to generate ICD-10 diagnoses of depressive episode and anxiety disorders (generalized anxiety disorder, panic disorder, phobia, obsessive-compulsive disorder) [37].

#### Social support

This was assessed with a 7-item measure. Using answer options ‘not true’ (score = 0), ‘partly true’ (score = 1) and ‘certainly true’ (score = 2), participants responded to statements which inquired if, family and friends did things to make them happy, made them feel loved, could be relied on no matter what, would see that they were taken care of no matter what, accepted them just the way they are, made them feel an important part of their lives, and gave them support and encouragement. Responses were added to create a scale score that could range from 0 to 14. The internal consistency of the scale was good: α = 0.89.

#### Socio-demographic variables

Information was also obtained on age, sex, equivalized income tertiles (high ≥£29826, middle £14,057 to < £29826, low <£14,057), education [qualification (degree, non-degree, A-level, GCSE, other): yes or no)], and ethnicity (white British or other).

### Statistical analysis

Differences in sample characteristics by the presence of loneliness were tested with Chi-square tests and Student’s *t*-tests for categorical and continuous variables, respectively. To examine the association between physical multimorbidity and loneliness, a multivariable logistic regression analysis was performed with loneliness as the dependent variable and the number of physical diseases as the independent variable that adjusted for the socio-demographic variables (age, sex, income, education, and ethnicity). An age-stratified analysis was also conducted to assess whether the association differed between different age groups.

To determine if any factors mediated the association between loneliness and physical multimorbidity (i.e., ≥2 physical diseases) (outcome), a meditation analysis was performed using the *khb* (Karlson Holm Breen) command in Stata [38]. This method can be used with logistic regression analysis to decompose the total effect of a variable into its direct and indirect effects (i.e., mediational effect). This allows the percentage of the main association explained by the mediator to be calculated (mediated percentage). Potential mediators (smoking, alcohol dependence, drug use, disordered eating, obesity, stressful life events, social support, depression, anxiety) were selected using past literature as a guide. Each potential mediator was included in the model individually. The mediation analysis controlled for the five socio-demographic variables. In order to assess whether the mediators differ by age group, we also conducted age-stratified analyses.

As nearly 20% of the participants had missing income information and in order to avoid the exclusion of a large number of respondents, a missing category for this variable was included in all regression analyses. Age-stratified analyses were not adjusted for age. All variables were included in the models as categorical variables, excepting the stressful life events and social support variables (continuous). The sample weighting and the complex study design were taken into account in order to generate nationally representative estimates. Odds ratios (OR) and 95% confidence intervals (95%CI) are reported. The level of statistical significance was set at P<0.05. All analyses were performed with Stata version 14.1 (Stata Corp LP, College Station, Texas).

## Results

**Fig 1:**
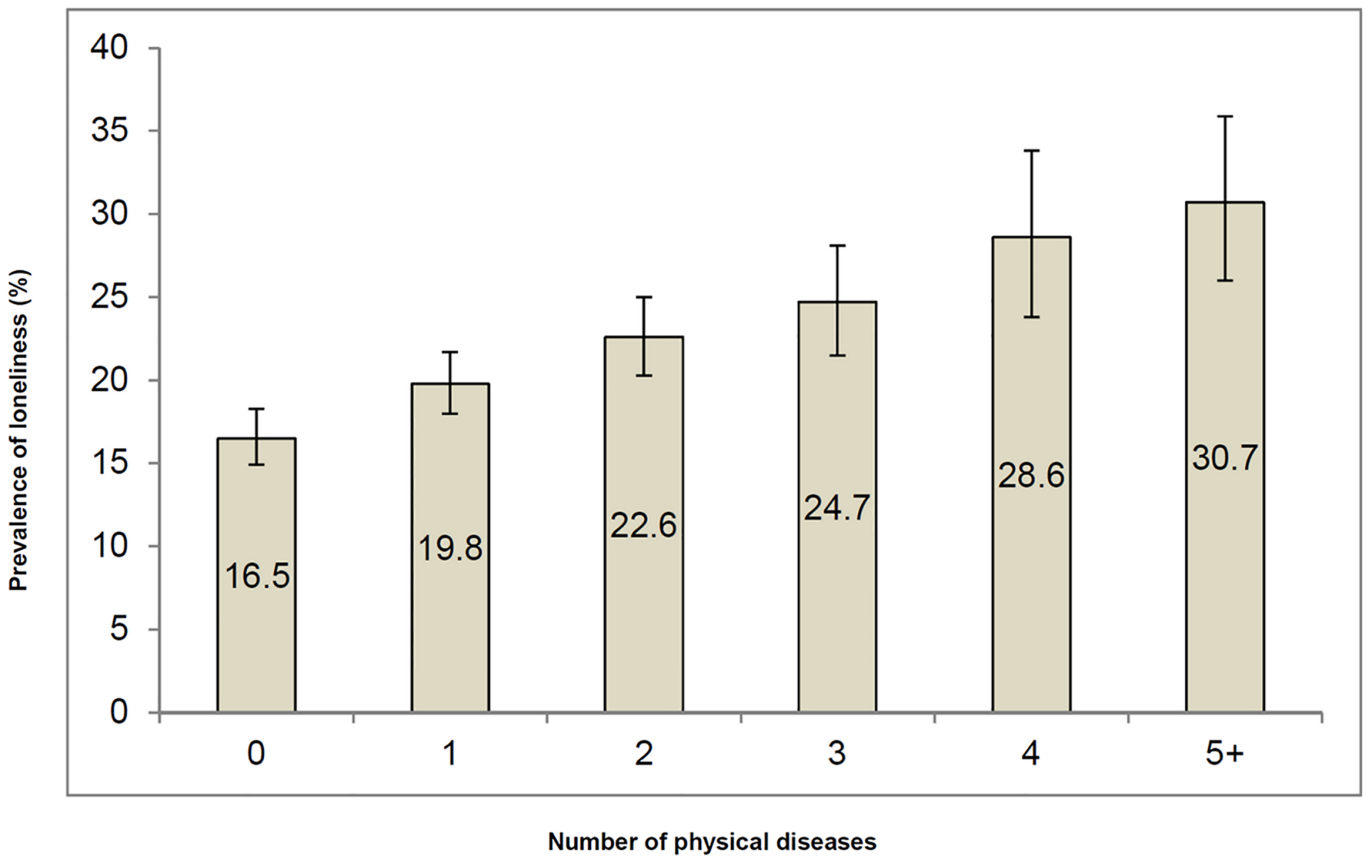
Prevalence of loneliness by the number of physical diseases. Bars denote 95% confidence intervals. Estimates are based on weighted sample.

There were significant differences between lonely and non-lonely people for all of the sample characteristics except for ethnicity and smoking status (Table 1). Lonely people were more likely to have two or more physical diseases. A graded association between the presence of physical disease and loneliness is illustrated in Fig 1 with the prevalence of loneliness rising from 16.5% in those with no physical diseases to 30.7% among those with 5 or more diseases. Besides a higher number of physical diseases, lonely people also had a higher prevalence of adverse outcomes such as alcohol dependence, drug use, stressful events across the life course, poorer mental health (anxiety and depression) and lower social support.

**Table 1:** Sample characteristics (overall and by loneliness).

Characteristic	Category	Overall		Loneliness				P-value^a^
				No		Yes		
Number of physical diseases	0	2,373	36.5	1,961	38.3	412	29.6	<**0.001**
	1	2,020	28.3	1,593	28.6	427	27.5	
	2	1,335	16.7	1,017	16.3	318	18.5	
	3	804	9.4	587	8.9	217	11.3	
	4	402	4.6	276	4.1	126	6.4	
	≥5	423	4.5	279	3.9	144	6.7	
Age (y)	16–44	2,871	48.1	2,183	47.0	688	52.4	<**0.001**
	45–64	2,406	32.3	1,864	32.6	542	31.2	
	≥65	1,954	19.6	1,564	20.4	390	16.5	
Sex	Male	3,176	48.6	2,544	50.1	632	42.8	<**0.001**
	Female	4,184	51.4	3,172	49.9	1,012	57.2	
Income	High	1,972	35.9	1,623	37.4	349	29.5	<**0.001**
	Middle	1,932	32.6	1,512	32.9	420	31.5	
	Low	1,957	31.5	1,426	29.7	531	39.0	
Qualification	No	2,103	23.9	1,570	23.3	533	26.1	**0.041**
	Yes	5,225	76.1	4,123	76.7	1,102	73.9	
Ethnicity	British White	6,478	85.2	5,053	85.5	1,425	84.1	0.285
	Other	847	14.8	639	14.5	208	15.9	
Smoking	No	2,481	34.7	1,971	35.3	510	32.2	0.339
	Yes	4,879	65.3	3,745	64.7	1,134	67.8	
Alcohol dependence	No	6,995	94.1	5,493	95.4	1,502	89.2	<**0.001**
	Yes	365	5.9	223	4.6	142	10.8	
Drug use	No	6,813	90.7	5,369	92.3	1,444	84.7	<**0.001**
	Yes	534	9.3	340	7.7	194	15.3	
Disordered eating	No	6,897	93.6	5,500	96.2	1,397	83.2	<**0.001**
	Yes	450	6.4	206	3.8	244	16.8	
Obesity class I (BMI≥35 kg/m^2^)	No	6,617	94.6	5,163	95.0	1,454	93.0	**0.009**
	Yes	388	5.4	280	5.0	108	7.0	
Stressful life events (≥16 years)^b^	Mean (SD)		2.6 (2.0)		2.5 (1.9)		3.3 (2.5)	<**0.001**
Stressful life events (<16 years)^c^	Mean (SD)		0.5 (1.0)		0.4 (0.9)		0.8 (1.2)	<**0.001**
Social support^d^	Mean (SD)		13.2 (1.9)		13.4 (1.6)		12.4 (2.8)	<**0.001**
Depression	No	7,109	97.0	5,651	99.0	1,458	89.3	<**0.001**
	Yes	251	3.0	65	1.0	186	10.7	
Anxiety	No	6,828	93.3	5,537	97.0	1,291	78.7	<**0.001**
	Yes	532	6.7	179	3.0	353	21.3	

*Note*: Boldface type indicates statistical significance (*p*<0.05).

Abbreviation: SD standard deviation; Data are unweighted N and weighted percentage or mean (SD).

^a^ The differences in sample characteristics was tested by Chi-square tests and Student’s t-tests for categorical and continuous variables respectively.

^b^ Based on 17 stressful life events occurring at the age of 16 or after.

^c^ Based on 8 stressful life events occurring before the age of 16.

^d^ Seven items were used to identify the level of social support with each item having scores of 0, 1, or 2. Scores of the 7 items were added to create a scale ranging from 0–14 with higher scores corresponding to higher levels of social support.

In a multivariable logistic regression analysis that controlled for socio-demographic factors, there was a statistically significant monotonic association between the number of physical diseases (independent variable) and loneliness (dependent variable) with the OR for loneliness rising from 1.34 (95% CI: 1.13–1.59) for those with one disease to almost 3 (OR: 2.82, 95% CI: 2.11–3.78) for those with 5 or more diseases, compared to those with no physical diseases. When the analysis was stratified by age, the associations were stronger across all levels of disease in the youngest age group (16–44 years) (Table 2).

**Table 2:** Association between number of physical diseases and loneliness estimated by multivariable logistic regression analysis.

	Overall		Age (16–44 years)		Age (45–64 years)		Age (≥65 years)	
Characteristics	OR	95%CI	OR	95%CI	OR	95%CI	OR	95%CI
**Number of physical diseases**								
0	1.00		1.00		1.00		1.00	
1	1.34***	**[1.13,1.59]**	1.41**	**[1.14,1.75]**	0.91	[0.61,1.37]	0.93	[0.61,1.43]
2	1.66***	**[1.37,2.02]**	1.84***	**[1.39,2.45]**	1.24	[0.82,1.89]	1.28	[0.83,1.97]
3	1.92***	**[1.52,2.43]**	2.24***	**[1.49,3.37]**	1.47	[0.96,2.23]	1.53	[0.99,2.35]
4	2.57***	**[1.92,3.43]**	2.97***	**[1.68,5.25]**	1.77*	**[1.09,2.89]**	1.78*	**[1.08,2.93]**
≥5	2.82***	**[2.11,3.78]**	3.09**	**[1.53,6.24]**	1.90**	**[1.23,2.93]**	1.94**	**[1.25,3.01]**
**Age** (years)								
16–44	1.00							
45–64	0.70***	**[0.61,0.82]**						
≥65	0.44***	**[0.37,0.53]**						
**Sex**								
Male	1.00		1.00		1.00		1.00	
Female	1.21**	**[1.07,1.37]**	1.16	[0.96,1.41]	1.73***	**[1.34,2.24]**	1.75***	**[1.34,2.28]**
**Income**								
High	1.00		1.00		1.00		1.00	
Middle	1.22*	**[1.03,1.44]**	1.09	[0.83,1.43]	1.49	[0.89,2.51]	1.51	[0.88,2.61]
Low	1.62***	**[1.37,1.91]**	1.65***	**[1.28,2.12]**	1.29	[0.77,2.15]	1.28	[0.74,2.21]
**Qualification**								
No	1.00		1.00		1.00		1.00	
Yes	0.84*	**[0.71,0.99]**	0.73	[0.52,1.04]	0.68**	**[0.54,0.86]**	0.67**	**[0.53,0.85]**
**Ethnicity**								
British White	1.00		1.00		1.00		1.00	
Other	1.06	[0.86,1.31]	1.07	[0.83,1.37]	1.46	[0.80,2.67]	1.44	[0.74,2.78]

*Note*: Boldface type indicates statistical significance (* *p*<0.05, ** *p*<0.01, *** *p*<0.001)

Abbreviation: OR odds ratio; CI confidence interval

Models are adjusted for all variables in the respective columns.

The mediation analysis showed that stressful life events both before and after 16 years of age mediated 11.1% and 30.5% of the association between loneliness and multimorbidity, respectively, while depression (15.4% mediated), anxiety (30.2%) and disordered eating (10.1%) were also important mediators. In contrast, there were no significant mediational effects for smoking, alcohol dependence, drug use, obesity or social support (Table 3). When an age-stratified mediation analysis was performed, the same variables were important for adults aged 16–44 and 45–64. Specifically, depression, anxiety, stressful life events before and after age 16 and disordered eating were all significant mediators in the association between multimorbidity and loneliness although stressful life events in adulthood was the strongest mediator for adults aged 16–44 whereas for adults aged 45–64, anxiety was the strongest mediator. For adults aged 65 and above the only variable which was a significant mediator was stressful life events in adulthood which mediated 11.8% of the association (S2 Table).

**Table 3:** Lifestyle and other potential mediators in the association between loneliness and multimorbidity.

Mediator	Effect	OR	95%CI	P-value	% Mediated
Smoking	Total	1.67	[1.46, 1.91]	<**0.001**	NA
	Direct	1.66	[1.46, 1.90]	<**0.001**	
	Indirect	1.01	[1.00, 1.01]	0.085	
Alcohol dependence	Total	1.67	[1.46, 1.91]	<**0.001**	NA
	Direct	1.65	[1.44, 1.88]	<**0.001**	
	Indirect	1.01	[1.00, 1.03]	0.078	
Drug use	Total	1.67	[1.46, 1.91]	<**0.001**	NA
	Direct	1.65	[1.44, 1.88]	<**0.001**	
	Indirect	1.01	[1.00, 1.03]	0.137	
Disordered eating	Total	1.67	[1.46, 1.91]	<**0.001**	10.1
	Direct	1.58	[1.38, 1.81]	<**0.001**	
	Indirect	1.05	[1.02, 1.08]	<**0.001**	
Obesity class I	Total	1.64	[1.44, 1.88]	<**0.001**	NA
(BMI≥35 kg/m^2^)	Direct	1.62	[1.42, 1.86]	<**0.001**	
	Indirect	1.01	[1.00, 1.03]	0.081	
Stressful life events	Total	1.68	[1.47, 1.93]	<**0.001**	30.5
(≥16 years)	Direct	1.44	[1.25, 1.65]	<**0.001**	
	Indirect	1.17	[1.13, 1.22]	<**0.001**	
Stressful life events	Total	1.67	[1.46, 1.91]	<**0.001**	11.1
(<16 years)	Direct	1.58	[1.38, 1.81]	<**0.001**	
	Indirect	1.06	[1.03, 1.09]	<**0.001**	
Social support	Total	1.67	[1.47, 1.91]	<**0.001**	NA
	Direct	1.68	[1.46, 1.93]	<**0.001**	
	Indirect	1.00	[0.97, 1.02]	0.806	
Depression	Total	1.67	[1.46, 1.91]	<**0.001**	15.4
	Direct	1.54	[1.35, 1.77]	<**0.001**	
	Indirect	1.08	[1.05, 1.12]	<**0.001**	
Anxiety	Total	1.67	[1.46, 1.91]	<**0.001**	30.2
	Direct	1.43	[1.25, 1.64]	<**0.001**	
	Indirect	1.17	[1.12, 1.22]	<**0.001**	

*Note*: Boldface type indicates statistical significance (*p*<0.05)

Abbreviation: OR odds ratio; CI confidence interval

Models are adjusted for age, sex, income, qualification, and ethnicity.

Multimorbidity refers to two or more physical diseases.

Percentage mediated is only provided when the indirect effect is statistically significant.

## Discussion

This study examined the association between multiple co-occurring physical diseases and loneliness in a nationally representative sample of the English population. The results showed that the number of co-occurring physical diseases was associated with loneliness in a dose-response fashion after adjustment for socio-demographic factors, particularly in the youngest age group (aged 16–44 years). In addition, stressful life events across the life course and poor mental health (anxiety and depression) were important mediators in the association between multimorbidity and loneliness.

Although prior research has produced mixed results on the association between the number of diseases/multimorbidity and loneliness [25–29], the results of the current study accord with previous studies which showed an association [25–28], and which also found that the strength of the association varied between different populations/population subgroups [26]. The exact way in which physical disease and loneliness are linked is uncertain although the results from the mediation analysis point to several possible pathways. In particular, mental illness was an important mediator in the association. This accords with research which has linked physical multimorbidity and depression [10], possibly due to the feelings of inadequacy, dependency and dejection it can give rise to [39], as well as with studies which have shown that worse mental health is associated with loneliness [40]. In terms of the specific association, a recent study found that depression was linked to lower participation in social leisure activities in older adults with multimorbidity [41] which might be important for feelings of loneliness.

Stressful life events across the life course (both before and after age 16) were also an important factor in the association between multimorbidity and loneliness. Research has shown that stressful events in early life are linked to an increased risk for both later multimorbidity and loneliness [42,43] possibly as a result of physiological changes they may cause that might increase the risk for future disease [44] as well as through problems with bonding and trust [43]. Given this, it is possible that such events might be a common third factor underlying the occurrence of both multimorbidity and loneliness in adulthood. Alternatively, stressful life events might also mediate the association with loneliness. For example, stressors such as divorce, separation and widowhood that might be important for loneliness have been associated with multimorbidity in middle-aged adults [45]. Another adult stressor which might be a direct consequence of co-occurring physical diseases—an inability to work—might also play a role here, as multimorbidity has been associated with unemployment [46] which in turn, might be important for loneliness [12,47].

As this study was cross-sectional it was not possible for us to establish causality or determine the direction of the observed associations. It is possible therefore that loneliness might also have led to multimorbidity. For instance, it has been suggested that the hypervigilance for social threats in the surrounding environment, distorted cognitions and a negative disposition (e.g. feelings of stress, anxiety, hostility) that are all associated with loneliness may result in behavioral and physiological changes that can impact negatively on health [48]. Indeed, there is a growing body of evidence that loneliness is linked to biological changes that might be adverse for health such as higher systolic blood pressure [49], differences in diastolic blood pressure reactivity [50] and cardiovascular functioning [51], as well as an increased risk for metabolic syndrome [52] that might underlie co-occurring physical diseases. Our finding that stressful life events link multimorbidity and loneliness also provides some support for the idea that loneliness might be a precursor of physical disease and multimorbidity as previous research has indicated that (perceived) stress may mediate the association between loneliness and physical ill health [53,54].

Stratifying the analysis by age revealed that the association between physical multimorbidity and loneliness was strongest in the youngest age group. Given that multimorbidity has been associated with increasing age [55] and that it has been hypothesized that the effects of loneliness might accrue across the life course in terms of their negative effect on physiological resilience (and thus, health) [56], the finding of a stronger association between multimorbidity and loneliness in the youngest age group is somewhat unexpected and it can only be speculated what underlies this result. It is possible, for instance, that a higher number of chronic diseases might affect a younger person’s ability to engage in age-related social roles (e.g., spouse, parent, and/or worker) and social activities and thus act to isolate them. In contrast, older individuals might have fewer roles or activities and/or had more time to adapt to the inhibiting effects of several physical illnesses so that only those with a higher number of co-occurring illnesses experience loneliness. Indeed, the potentially greater detrimental impact of co-occurring diseases at a younger age might explain why depression and anxiety were important mediators in the association between multimorbidity and loneliness in those aged under 65 but not in the oldest age group. The same might also be true when looking at loneliness leading to physical disease. Specifically, as research has indicated that the prevalence of loneliness might be higher at both ends of the life span i.e. in adolescence/young adulthood and old age [57], it is possible that loneliness might have a stronger effect on well-being during an age period where people are expected to be involved in a variety of personal and work-related relations and where loneliness is less common.

This study has several limitations. Physical illnesses were self-reported and were not verified against other sources of data such as medical records. This might have resulted in misreporting in some instances especially as evidence suggests that while self-reports of physical illness mostly accord with doctor’s reports, not all diseases are reported with equal accuracy, and there might also be age-related differences in the reliability of reports for some diseases [58]. In addition, the survey response rate was moderate (57%) [30]. As research has shown that lonely individuals trust others less and may be less likely to disclose intimate information for fear that confidentiality will not be maintained [59], it is possible that they might have been over-represented among non-responders which might have affected the results (i.e. non-response bias). Finally, following the lead of an earlier study which used APMS data [33], we used an item from the SFQ as a measure of loneliness. However, it should be noted that this item also mentioned ‘isolation’. We were unable therefore to tease apart the association between loneliness and social isolation in our study despite the fact that they are often studied separately and may have different effects on health outcomes [60].

In conclusion, this study has shown that physical multimorbidity is associated with increased odds for loneliness. Given the possible bi-directionality of the association between ill health and loneliness, this highlights the importance of future longitudinal research to better specify the association between these phenomena across time, and the potential mechanisms that might underlie it.

## Supporting information

S1 Table Stressful life events. Events that occurred before the age of 16 are indicated with a tick mark.
(DOCX)

S2 Table Lifestyle and other potential mediators in the association between loneliness and multimorbidity (by age group). (DOCX)
