# Supplementary material for: Physical multimorbidity and loneliness: A population-based study
Source: PLoS One. 2018 Jan 24;13(1):e0191651. doi: 10.1371/journal.pone.0191651 (PMC5783394; doi:10.1371/journal.pone.0191651)
Supplement: S1 Table — Events that occurred before the age of 16 are indicated with a tick mark. (DOCX) [file pone.0191651.s001.docx]

| **S1 Table List of stressful life events** | | |
| --- | --- | --- |
|  |  | <16 years |
| 1 | Serious illness, injury or assault to a close relative | 🗸 |
| 2 | Death of an immediate family member of yours | 🗸 |
| 3 | Death of a close family friend or other relative, like an aunt, cousin or grandparent | 🗸 |
| 4 | Separation due to marital difficulties, divorce or steady relationship broken down | 🗸 |
| 5 | Serious problem with a close friend, neighbor or relative |  |
| 6 | Being made redundant or sacked from your job |  |
| 7 | Looking for work without success for more than 1 month |  |
| 8 | Major financial crisis, like losing the equivalent of 3 months income |  |
| 9 | Problem with police involving court appearance |  |
| 10 | Something you valued being lost or stolen |  |
| 11 | Bullying | 🗸 |
| 12 | Violence at work |  |
| 13 | Violence in the home | 🗸 |
| 14 | Sexual abuse | 🗸 |
| 15 | Being expelled from school |  |
| 16 | Running away from your home | 🗸 |
| 17 | Being homeless |  |
