# Supplementary material for: Physical multimorbidity and loneliness: A population-based study
Source: PLoS One. 2018 Jan 24;13(1):e0191651. doi: 10.1371/journal.pone.0191651 (PMC5783394; doi:10.1371/journal.pone.0191651)
Supplement: S2 Table — (DOCX) [file pone.0191651.s002.docx]

| **S2 Table** Lifestyle and other potential mediators in the association between loneliness and multimorbidity (by age group) | | | | | | | | | | | | | |
| --- | --- | --- | --- | --- | --- | --- | --- | --- | --- | --- | --- | --- | --- |
|  |  | Age 16-44 years | | | | Age 45-64 years | | | | Age ≥65 years | | | |
| Mediator | Effect | OR | 95%CI | P-value | % Mediated | OR | 95%CI | P-value | % Mediated | OR | 95%CI | P-value | % Mediated |
| Smoking | Total | 1.82 | [1.46, 2.25] | **<0.001** | NA | 1.62 | [1.30, 2.01] | **<0.001** | NA | 1.63 | [1.26, 2.10] | **<0.001** | NA |
|  | Direct | 1.79 | [1.44, 2.22] | **<0.001** |  | 1.62 | [1.30, 2.01] | **<0.001** |  | 1.62 | [1.26, 2.09] | **<0.001** |  |
|  | Indirect | 1.01 | [1.00, 1.03] | 0.099 |  | 1.00 | [1.00, 1.01] | 0.776 |  | 1.01 | [0.99, 1.02] | 0.417 |  |
| Alcohol dependence | Total | 1.81 | [1.46, 2.24] | **<0.001** | NA | 1.62 | [1.30, 2.01] | **<0.001** | NA | 1.63 | [1.26, 2.10] | **<0.001** | NA |
|  | Direct | 1.75 | [1.41, 2.18] | **<0.001** |  | 1.61 | [1.30, 2.00] | **<0.001** |  | 1.63 | [1.26, 2.10] | **<0.001** |  |
|  | Indirect | 1.03 | [1.00, 1.07] | 0.050 |  | 1.00 | [0.98, 1.03] | 0.710 |  | 1.00 | [0.99, 1.01] | 0.616 |  |
| Drug use | Total | 1.81 | [1.46, 2.24] | **<0.001** | NA | 1.62 | [1.30, 2.01] | **<0.001** | NA | 1.62 | [1.26, 2.10] | **<0.001** | NA |
|  | Direct | 1.77 | [1.43, 2.19] | **<0.001** |  | 1.61 | [1.30, 2.00] | **<0.001** |  | 1.62 | [1.26, 2.10] | **<0.001** |  |
|  | Indirect | 1.02 | [0.99, 1.06] | 0.193 |  | 1.00 | [0.99, 1.01] | 0.553 |  | 1.00 | [1.00, 1.01] | 0.807 |  |
| Disordered eating | Total | 1.80 | [1.45, 2.23] | **<0.001** | 14.7 | 1.62 | [1.31, 2.02] | **<0.001** | 8.6 | 1.62 | [1.25, 2.08] | **<0.001** | NA |
|  | Direct | 1.65 | [1.32, 2.06] | **<0.001** |  | 1.56 | [1.25, 1.94] | **<0.001** |  | 1.62 | [1.26, 2.09] | **<0.001** |  |
|  | Indirect | 1.09 | [1.03, 1.15] | **0.002** |  | 1.04 | [1.00, 1.09] | **0.043** |  | 1.00 | [0.99, 1.01] | 0.672 |  |
| Obesity class I | Total | 1.74 | [1.39, 2.18] | **<0.001** | NA | 1.64 | [1.32, 2.05] | **<0.001** | NA | 1.62 | [1.25, 2.10] | **<0.001** | NA |
| (BMI≥35 kg/m^2^) | Direct | 1.72 | [1.38, 2.16] | **<0.001** |  | 1.61 | [1.29, 2.01] | **<0.001** |  | 1.60 | [1.23, 2.07] | **<0.001** |  |
|  | Indirect | 1.01 | [0.99, 1.03] | 0.347 |  | 1.02 | [1.00, 1.05] | 0.111 |  | 1.01 | [0.99, 1.03] | 0.325 |  |
| Stressful life events | Total | 1.82 | [1.47, 2.26] | **<0.001** | 41.8 | 1.64 | [1.32, 2.04] | **<0.001** | 27.3 | 1.65 | [1.27, 2.13] | **<0.001** | 11.8 |
| (≥16 years) | Direct | 1.42 | [1.14, 1.77] | **0.002** |  | 1.43 | [1.15, 1.79] | **0.002** |  | 1.55 | [1.20, 2.01] | **0.001** |  |
|  | Indirect | 1.29 | [1.20, 1.38] | **<0.001** |  | 1.14 | [1.08, 1.21] | **<0.001** |  | 1.06 | [1.02, 1.10] | **0.004** |  |
| Stressful life events | Total | 1.81 | [1.45, 2.25] | **<0.001** | 17.4 | 1.62 | [1.30, 2.02] | **<0.001** | 9.0 | 1.63 | [1.26, 2.10] | **<0.001** | NA |
| (<16 years) | Direct | 1.63 | [1.30, 2.05] | **<0.001** |  | 1.55 | [1.25, 1.93] | **<0.001** |  | 1.62 | [1.26, 2.10] | **<0.001** |  |
|  | Indirect | 1.11 | [1.05, 1.17] | **<0.001** |  | 1.04 | [1.01, 1.08] | **0.008** |  | 1.00 | [0.99, 1.02] | 0.765 |  |
| Depression | Total | 1.79 | [1.44, 2.23] | **<0.001** | 14.0 | 1.63 | [1.31, 2.03] | **<0.001** | 19.3 | 1.64 | [1.27, 2.12] | **<0.001** | NA |
|  | Direct | 1.65 | [1.33, 2.06] | **<0.001** |  | 1.48 | [1.19, 1.86] | **0.001** |  | 1.57 | [1.22, 2.02] | **<0.001** |  |
|  | Indirect | 1.09 | [1.03, 1.14] | **0.001** |  | 1.10 | [1.04, 1.17] | **0.002** |  | 1.04 | [0.99, 1.10] | 0.096 |  |
| Anxiety | Total | 1.77 | [1.42, 2.21] | **<0.001** | 34.0 | 1.64 | [1.31, 2.05] | **<0.001** | 33.9 | 1.63 | [1.27, 2.10] | **<0.001** | NA |
|  | Direct | 1.46 | [1.15, 1.85] | **0.002** |  | 1.39 | [1.11, 1.74] | **0.005** |  | 1.56 | [1.21, 2.01] | **0.001** |  |
|  | Indirect | 1.21 | [1.13, 1.30] | **<0.001** |  | 1.18 | [1.10, 1.27] | **<0.001** |  | 1.05 | [0.99, 1.11] | 0.091 |  |
| Social support | Total | 1.81 | [1.46, 2.24] | **<0.001** | NA | 1.63 | [1.32, 2.03] | **<0.001** | NA | 1.62 | [1.26, 2.09] | **<0.001** | NA |
|  | Direct | 1.74 | [1.40, 2.18] | **<0.001** |  | 1.69 | [1.35, 2.12] | **<0.001** |  | 1.64 | [1.26, 2.12] | **<0.001** |  |
|  | Indirect | 1.04 | [0.99, 1.08] | 0.114 |  | 0.96 | [0.92, 1.01] | 0.111 |  | 0.99 | [0.95, 1.04] | 0.723 |  |

*Note*: Boldface type indicates statistical significance (*p*<0.05)

Abbreviation: OR odds ratio; CI confidence interval

Models are adjusted for sex, income, qualification, and ethnicity.

Multimorbidity refers to two or more physical diseases.

Percentage mediated is only provided when the indirect effect is statistically significant.
